# A Glitch in the Matrix: The Role of Extracellular Matrix Remodeling in Opioid Use Disorder

**DOI:** 10.3389/fnint.2022.899637

**Published:** 2022-06-09

**Authors:** Madelyn H. Ray, Benjamin R. Williams, Madeline K. Kuppe, Camron D. Bryant, Ryan W. Logan

**Affiliations:** ^1^Laboratory of Sleep, Rhythms, and Addiction, Department of Pharmacology and Experimental Therapeutics, Boston University School of Medicine, Boston, MA, United States; ^2^Whitaker Cardiovascular Institute, Boston University School of Medicine, Boston, MA, United States; ^3^Center for Systems Neuroscience, Boston University, Boston, MA, United States; ^4^Laboratory of Addiction Genetics, Department of Pharmacology and Experimental Therapeutics, Boston University School of Medicine, Boston, MA, United States; ^5^Department of Psychiatry, Boston University School of Medicine, Boston, MA, United States; ^6^Genome Science Institute, Boston University School of Medicine, Boston, MA, United States

**Keywords:** opioids, extracellular matrix, addiction, neuroinflammation, opioid use disorder (OUD), sex differences

## Abstract

Opioid use disorder (OUD) and deaths from drug overdoses have reached unprecedented levels. Given the enormous impact of the opioid crisis on public health, a more thorough, in-depth understanding of the consequences of opioids on the brain is required to develop novel interventions and pharmacological therapeutics. In the brain, the effects of opioids are far reaching, from genes to cells, synapses, circuits, and ultimately behavior. Accumulating evidence implicates a primary role for the extracellular matrix (ECM) in opioid-induced plasticity of synapses and circuits, and the development of dependence and addiction to opioids. As a network of proteins and polysaccharides, including cell adhesion molecules, proteases, and perineuronal nets, the ECM is intimately involved in both the formation and structural support of synapses. In the human brain, recent findings support an association between altered ECM signaling and OUD, particularly within the cortical and striatal circuits involved in cognition, reward, and craving. Furthermore, the ECM signaling proteins, including matrix metalloproteinases and proteoglycans, are directly involved in opioid seeking, craving, and relapse behaviors in rodent opioid models. Both the impact of opioids on the ECM and the role of ECM signaling proteins in opioid use disorder, may, in part, depend on biological sex. Here, we highlight the current evidence supporting sex-specific roles for ECM signaling proteins in the brain and their associations with OUD. We emphasize knowledge gaps and future directions to further investigate the potential of the ECM as a therapeutic target for the treatment of OUD.

## Introduction

In the United States, rates of opioid use disorder (OUD) and deaths from overdose have continued to climb over recent years, particularly in adolescents and young adults. Accompanying a rise in deaths from drug overdose has been a steady increase in the number of people diagnosed with OUD. Current estimates reflect more than 3 million people have OUD, with an estimated 200,000 new diagnoses annually. Despite the enormous public health impact of OUD, we lack a basic understanding of the neurobiological mechanisms that contribute to OUD and the associated health consequences. OUD is a chronic, relapsing brain disease that can be managed by long-term medical interventions and maintenance therapies such as methadone or buprenorphine. Yet, ∼90–95% of people with OUD relapse despite treatment, as cravings and other challenges such as protracted withdrawal, persist for weeks, months, and years (Smyth et al., 2010; Kadam et al., 2017; Montiel Ishino et al., 2020). Discovering new interventions and therapeutics for the treatment of OUD will require massive, parallel efforts, across multiple clinical and basic research domains. A critical effort will be necessary to further define the diverse array of consequences of chronic opioid use on the brain and body, along with an in-depth investigation into the cellular and molecular mechanisms in the brain involved in opioid reward, craving, and relapse.

Opioids lead to long-lasting changes in gene transcription, protein signaling, receptor activity, synaptic morphology and plasticity, as well as neural circuit function that contribute to the development of addiction (Hearing, 2019; Li et al., 2019; Madayag et al., 2019; Song et al., 2019; Valentinova et al., 2019; Koob, 2020; Jiang et al., 2021; Seney et al., 2021; Tavares et al., 2021; Trieu et al., 2022; Xue et al., 2022). A major class of signaling proteins involved in opioid-induced neural plasticity, include cell adhesion molecules (CAMs), matrix metalloproteinases (MMPs), and proteoglycans, and these proteins provide structural support to neurons, astrocytes, microglia in the formation of the extracellular matrix (ECM) and perineuronal nets (PNNs). ECM signaling proteins are involved in neurotransmission, synaptic plasticity, and vascular integrity in the brain. Over the recent decade, the ECM has become a focus as a major contributor to long-lasting neuroadaptations accompanying various processes, including learning, stress, and opioid use disorder.

## Role for Extracellular Matrix Signaling Proteins in Opioid Use Disorder

In the brain, the ECM is critical in the regulation of synaptic function, blood-brain barrier integrity, and cell-to-cell communication. The scaffolding of the ECM comprises polysaccharides and glycoproteins that provide the necessary structure to support communication between neurons, astrocytes, and microglia, and helps facilitate both the formation of new synapses and tuning of synaptic functions (Dityatev et al., 2010; Ferrer-Ferrer and Dityatev, 2018). In particular, the ECM signaling proteins, MMPs, are implicated in opioid reward and addiction (Ishiguro et al., 2006). MMPs are multifunctional proteases involved in a variety of cellular pathways and processes including inflammation, cell migration, and angiogenesis (Visse and Nagase, 2003).

**FIGURE 1:**
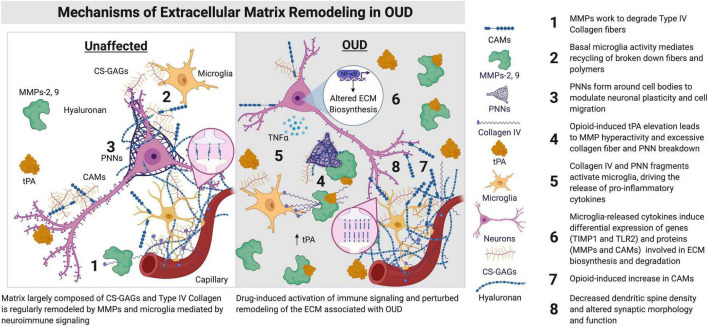
Synaptic morphology and function are regulated by ECM signaling proteins and microglia. The components of the ECM lie proximal to brain capillaries and vessels, condensed as PNNs around cell bodies, including neurons, astrocytes, and microglia, along with synapses and dendrites of neurons. ECM components are also distributed amongst cells of the brain within the parenchyma. Hyaluronan is primarily located in the neural interstitial matrix of the parenchyma. Hyaluronan is involved in the regulation of inflammation and myelination in the brain, including remyelination after insult or injury. Opioids lead to an increase in neuroimmune activation by microglia and other immune cell types in the brain. An induction of immune activation in the brain can lead to increased expression and activity of tPAs, MMPs, CAMs, and Collagen IV (Webersinke et al., 1992; Roberts et al., 2018). Augmented activity of these ECM signaling proteins remodels the ECM, with consequences on dendritic spine morphology, including the reduction of spine number in key regions associated with OUD (e.g., prefrontal cortex and nucleus accumbens). CAMs, cell adhesion molecules; CS-GAGs, chondroitin sulfate glycosaminoglycans; ECM, extracellular matrix; MMPs, matrix metalloproteinases; NF-κB, nuclear factor kappa B; OUD, opioid use disorder; PNNs, perineuronal nets; TIMP, tissue inhibitor of metalloprotease; TIMP1, TIMP metallopeptidase inhibitor 1; TLR2, toll-like receptor 2; tPA, type plasminogen activator. Figure created using BioRender.

Opioids likely augment the activity of MMPs in the brain, substantially remodeling the ECM, potentially leading to opioid-induced changes in astrocyte-neuronal communication, synaptic plasticity, and trafficking of excitatory receptors (Figure 1; Michaluk et al., 2009, 2011; Huntley, 2012). For example, opioids lead to increased expression of both MMP-2 and MMP-9 in cell lines (Gach et al., 2011), and notably, in the rodent (Chioma et al., 2021), and human (Kovatsi et al., 2013; Seney et al., 2021) brain. Both MMP-2 and MMP-9-dependent signaling may be important for opioid-induced degradation in the integrity of the blood-brain barrier and an increase in neuroinflammation associated with OUD in the human brain (Huntley, 2012; Dal-Pizzol et al., 2013; Song et al., 2015; Rempe et al., 2016; Hannocks et al., 2019; Seney et al., 2021; Akol et al., 2022). Indeed, OUD is associated with alterations in ECM signaling and dopaminergic, GABAergic, and opioidergic neurotransmission, along with increased neuroinflammation in the human dorsolateral prefrontal cortex and nucleus accumbens (Seney et al., 2021), major neural substrates for cognition, impulsivity, and reward. Consistent with this, intravenous self-administration of heroin leads to elevated activity of MMP-2 and MMP-9 in the nucleus accumbens of both male and female rats (Chioma et al., 2021). Notably, MMP activity returns to below baseline levels following extinction of heroin self-administration behavior (Chioma et al., 2021). Opioid-induced increases in MMP activity are preferential to dendritic spines of dopamine receptor 1-expressing (D1+) medium spiny neurons (Chioma et al., 2021). In D1+ medium spiny neurons, MMP-9 activity seems to be acutely upregulated by heroin, returning to control levels after the removal of the drug and/or drug-cue (Chioma et al., 2021). As one of the major cell types in the nucleus accumbens that regulates drug reward-related behaviors, D1+ medium spiny neurons and associated MMP activity may serve as a key mechanism in the response to both opioid-induced and context-dependent neural plasticity (Smith et al., 2014). In mice, opioid administration also increases MMP-9 activity to modulate dopaminergic neurotransmission from the ventral tegmental area to nucleus accumbens (Nakamoto et al., 2012).

Changes in MMP-2 and MMP-9 have been found in the blood from people being treated for morphine dependency (Najafi et al., 2018). While MMP-2 activity is increased in the serum of morphine-dependent patients, MMP-9 activity is decreased (Najafi et al., 2018). Other studies report elevated MMP-9 in blood from patients with OUD during opioid withdrawal (Salarian et al., 2018). Interestingly, both studies suggest MMP-9 reflects a possible treatment response, as the expression and activity of the MMP-9 are reduced by methadone therapy (Salarian et al., 2018) and other treatments (Najafi et al., 2018). Changes in MMP expression in the blood of patients being treated for opioid dependency and addiction may reflect functional alterations in the central nervous system that are critical in the development of tolerance and physical dependence. For example, MMP-9 is increased in the brain and spinal cord of mice administered morphine across multiple days and contributes to the development of morphine tolerance for nociception (Nakamoto et al., 2012) and physical dependence (Liu et al., 2010). Pharmacological blockade of MMP activity or knockout of MMP-9 prevents the development of morphine tolerance for nociception (Nakamoto et al., 2012). Morphine-induced upregulation of MMP-2 and MMP-9 production has been indicated in ECM maintenance, particularly as it pertains to type IV collagen degradation and recycling (Gach et al., 2012). Specifically, opioid-induced alterations in MMP-2 activity are driven by the nitric oxide/nitric oxide synthase (NO/NOS) system, which in turn is regulated by receptor families independent of the μ-opioid receptor, thereby indicating a need for further research in opioid receptor crosstalk and subsequent downstream signaling cascades. Of note, NO/NOS-related mechanisms are involved in opioid-induced inhibition of MMP-9 activity in an opioid-receptor-dependent manner (Gach et al., 2012). Therefore, ECM protein levels in the context of opioid use are tightly regulated by mechanisms dependent and independent of opioid receptor activity and are intertwined with the NO/NOS system. Taken together, these findings suggest that increases in MMP-2 and MMP-9 expression following opioid administration may be critical for behavioral tolerance and dependence as well as drug- and context-induced neural plasticity. Future studies should examine MMP-2 and MMP-9 in preclinical addiction model behaviors to examine their validity as potential therapeutic targets.

A subset of MMPs, including MMP-2 and MMP-9, are activated by the serine protease tissue-type plasminogen activator (tPA), a key regulator of drug-induced synaptic plasticity and remodeling in major reward pathways of the brain (Calabresi et al., 2000; Sternlicht and Werb, 2001; Samson and Medcalf, 2006). Opioid administration leads to increases in tPA levels in the prefrontal cortex, hippocampus, and nucleus accumbens (Nagai et al., 2004). Importantly, the increases in tPA and MMPs during opioid administration are critical for the development of opioid tolerance (Yan et al., 2007; Nakamoto et al., 2012). tPA is also involved in locomotor sensitization to morphine (Bahi and Dreyer, 2008) and regulates the acquisition and maintenance of morphine self-administration behaviors (Yan et al., 2007), presumably via the modulation of dopamine neurotransmission in the striatum (Nagai et al., 2004; Yan et al., 2007). While increases in tPA and MMP are consistently found following opioid administration (Figure 1), the specific roles of tPA and MMP in opioid seeking, craving, and relapse behaviors, as related to OUD are unknown, requiring more studies into the potential crosstalk between tPA and MMP pathways in brain and behavioral plasticity associated with chronic opioid use.

Another class of ECM proteins called cell adhesion molecules (CAMs) may be involved in opioid reward-related behaviors and OUD. CAMs facilitate interactions between the ECM and various cell types in the brain. CAMs bind to other cell adhesion proteins and neighboring neurons to regulate neuronal growth, synaptic plasticity, and function. In the brain, some of the more common CAMs include neural CAM (NCAM), and the Cadherin family, including cadherin-2 (CDH-2) (Polanco et al., 2021). In the hippocampus, knockdown of neural CAMs (NCAMs) decreases the formation of conditioned place preference to morphine (Ishiguro et al., 2006). Following a lethal dose of heroin, levels of NCAMs are increased in the hippocampus of postmortem brains from people with heroin addiction, which positively correlate with blood levels of heroin at the time of death (Weber et al., 2006). Levels of CDH-2 in peripheral plasma have been shown to be a potential biomarker for methadone treatment outcome, correlating with treatment success (Liu et al., 2020), while hippocampal RNA expression of CDH-2 is increased following oxycodone self-administration (Zhang et al., 2015). However, this effect is specific to adult, but not adolescent mice, suggesting developmental stage may moderate the role of CAMs in opioid self-administration. Thus, opioids may lead to rapid increases in CAMs in a dose- and age-dependent manner in the brain, although whether CAMs directly contribute to neuroadaptations associated with OUD is still unknown, as these changes could be due to the acute effects of opioids. Future studies should examine the specific nature of CAM interactions concerning opioid use and relapse, with a specific focus on NCAMs and CDH-2 as potential biomarkers of opioid use.

## An Interplay Between the Extracellular Matrix, Microglia, and Neuroinflammation in Opioid Use Disorder

The ECM, in conjunction with microglia and astrocytes, is integral in both pro- and anti-inflammatory responses in the brain. Several lines of evidence link pro-inflammatory cytokine signaling and microglial activity to susceptibility to opioid craving and reward processing (Bland et al., 2009; Hofford et al., 2019). Consistent with this, a recent study from our research group identified significant alterations in transcripts enriched for neuroinflammatory and ECM signaling in the dorsolateral prefrontal cortex and nucleus accumbens of people with OUD (Seney et al., 2021). For example, transcripts that are upregulated in both brain regions of people with OUD are enriched for tumor necrosis factor alpha (TNF-α) signaling via positive regulation of nuclear factor kappa B (NF-κB) (Seney et al., 2021). This finding further supports NF-κB-dependent activation of pro-inflammatory TNF-α signaling associated with OUD. While neuroinflammation may play a distinct role in OUD, of particular importance is the impact of neuroinflammatory cytokine signaling on ECM remodeling activity. In human and rodent brain, chondroitin sulfate glycosaminoglycans (CS-GAGs) accumulate around the synapse in response to inflammation (Li et al., 2013) and may be increased following chronic opioid use in human brain (Seney et al., 2021). Indeed, the CS-GAG pathway is enriched in both the dorsolateral prefrontal cortex and nucleus accumbens of people with OUD (Seney et al., 2021). This poses the possibility that opioids and/or withdrawal from opioids leads to the aggregation of CS-GAGs at the synapses of neurons in regions involved in cognition and reward processing in response to alterations in the homeostatic regulation of inflammatory activity (Figure 1).

Other factors involved in ECM signaling may also contribute to opioid reward-related behaviors and could be associated with OUD. For example, both TIMP metallopeptidase inhibitor 1 (TIMP1) and toll-like receptor 2 (TLR2) are involved in the remodeling of the ECM via inhibition of MMPs (Visse and Nagase, 2003; Ries, 2014) and were recently identified as hub genes (i.e., highly connected gene) within gene networks in the nucleus accumbens that were specifically associated with OUD (Seney et al., 2021). Possibly, chronic opioid use accompanied by periods of withdrawal induce the release of pro-inflammatory cytokines, in turn activating TLR2 and TIMP1, leading to remodeling of the ECM and altering synaptic plasticity and function. Activation of pro-inflammatory cascades by opioids are likely regulated by microglia, as cell type specific enrichment of markers demonstrate a potential primary role for microglia associated with OUD in the dorsolateral prefrontal cortex and nucleus accumbens (Seney et al., 2021). Notably, the same study found enrichment of integrin signaling pathways in OUD, suggesting integrins could be involved in the migration of microglia and/or the adherence of the ECM to microglia and neurons. Collectively, these findings provide strong support for the involvement of the ECM and microglia-dependent neuroinflammation (Shen et al., 2022) in OUD. Future studies combining new single nuclei sequencing technologies with histochemical approaches will be critical for further investigating the potential role of microglia and other cell types in inflammation and ECM remodeling related to OUD in the human brain.

Other studies provide additional support for an important, functional role of microglia in OUD. Pharmacological inhibition of microglia (e.g., via AV411 compound, minocycline, or ibudilast) in rodent models reduces opioid seeking and reward-related behaviors and attenuates the subjective measures of opioid withdrawal in humans (Hutchinson et al., 2008, 2009; Bland et al., 2009; Schwarz et al., 2011; Arezoomandan et al., 2016; Cooper et al., 2016; Pan et al., 2016). Reactivity of microglia to opioids may depend on “off-target” binding of opioid metabolites (e.g., morphine-3-glucuronide) to the toll-like receptor 4 (TLR4), initiating intracellular cascades involved in pro-inflammatory cytokine release and activation of the canonical NF-κB pathway that regulate opioid reward-related and analgesia behaviors (Zhang et al., 2017, 2020; Green et al., 2022). For example, opioid-induced hyperalgesia and the development of tolerance depends on the release of the cytokine interleukin-33 (IL-33) by astrocytes in the brain and spinal cord (Hu et al., 2021). The release of IL-33 into the extracellular space activates astrocytes and microglia through NF-κB-dependent signaling (Molofsky et al., 2015), and is recently identified as a modulator of microglial-dependent degradation of the ECM (Nguyen et al., 2020). Therefore, IL-33 is one molecular intermediary by which microglia induce ECM remodeling and promote synaptic plasticity in an experience-dependent manner. Other pro-inflammatory cytokines may also be involved in opioid-induced synaptic and behavioral plasticity, including TNF and interferon alpha (Wang et al., 2018; Hofford et al., 2019; Seney et al., 2021).

In addition to cytokines, specific substrates of the ECM are involved in microglial activation and neuronal functions related to opioid actions. The formation and integrity of perisynaptic ECM scaffolds and PNNs are regulated by microglia (Crapser et al., 2020a,b, 2021; Strackeljan et al., 2021). PNNs are located proximal to both neurons and glial cells, and in some cortical regions of the brain form dense nets that surround GABAergic interneurons (Kosaka and Heizmann, 1989; Brückner et al., 1993; Sorg et al., 2016; Jorgensen, 2021). In rats, PNNs are significantly reduced in the medial prefrontal cortex and nucleus accumbens following extinction from heroin operant self-administration behavior (Van den Oever et al., 2010). Specifically, the ECM proteins, tenascin-R (TNR) and brevican (BCAN) are downregulated during heroin abstinence, yet are upregulated in the medial prefrontal cortex and nucleus accumbens in response to cue-induced reinstatement (Van den Oever et al., 2010). TNR and BCAN are preferentially expressed in PNNs that surround GABAergic interneurons in the medial prefrontal cortex and nucleus accumbens. Following reinstatement heroin-seeking behavior, these GABAergic interneurons displayed elevated spiking activity and enhanced inhibition of pyramidal neurons in the medial prefrontal cortex (Van den Oever et al., 2010). Therefore, TNR and BCAN may be key proteins of the ECM signaling pathways involved in the function of PNNs that modulate GABAergic cell activity during opioid reward-related behaviors, long-term abstinence from opioids, and potentially involved in opioid craving and relapse (Van den Oever et al., 2010; Xue et al., 2014; Favuzzi et al., 2017; Roura-Martínez et al., 2020). Taken together, there is a complex interplay between microglia, ECM, and neuroinflammation, and further studies examining these interactions related to OUD could be valuable for identifying new approaches for developing effective therapeutics.

## Future Investigations Into Biological Sex as a Potential Mediator of Extracellular Matrix Remodeling and Synaptic Plasticity in Opioid Use Disorder

Susceptibility to OUD and the severity of the related symptoms are the result of a complex interplay of biological and psychosocial factors. Earlier studies describe sex-specific differences in the frequency of use of opioids and the prevalence of clinical diagnoses of OUD. For example, higher rates of opioid use and OUD have been reported in men compared to women (Lee and Ho, 2013), although women may have accelerated progression from initial use to dependence (Kosten et al., 1993; Brady and Randall, 1999). Additionally, risk and frequency of opioid overdoses and propensity to use heroin has been described in men compared to women, while women may be more likely to misuse prescription opioids (Parlier-Ahmad et al., 2021). Comorbid psychiatric disorders, such as major depression, are also more prevalent in women compared to men diagnosed with OUD (Parlier-Ahmad et al., 2021).

Preclinical rodent models of opioid-related behaviors support sex-specific effects in opioid seeking, craving, and relapse, along with opioid withdrawal. Indeed, female rats tend to acquire morphine or heroin self-administration behavior quicker and display higher motivation to self-administer opioids compared to males (Lynch and Carroll, 1999; Cicero et al., 2003; Roth et al., 2004). Oxycodone self-administration is also significantly greater in female than male rats for both oral (Sharp et al., 2021) and intravenous self-administration (Kimbrough et al., 2020). Further, female rats exhibit higher sensitivity to the rewarding effects of morphine at far lower doses (Karami and Zarrindast, 2008). During withdrawal from opioids, female and male rats exhibit similarly elevated somatic symptoms (i.e., foot licks, grooming, and writhing) for nearly 48 h following opioid cessation (Gipson et al., 2021). Only increased body temperature was specific to female rats during withdrawal relative to males (Gipson et al., 2021). However, other studies report exaggerated somatic opioid withdrawal symptoms in both the severity and duration in male rodents (Cicero et al., 2002; Diaz et al., 2005). Activity of mu and/or kappa opioid receptors may also be involved in sex-specific effects of opioids (Barrett et al., 2002; Negus et al., 2002). Given this evidence highlighting sex as a critical factor in OUD and opioid actions, more studies are required to investigate the sex-specific cellular and molecular mechanisms involved in opioid reward, treatment response to opioids, and the development of dependence and tolerance to opioids. Few studies have directly examined the role of sex in ECM signaling in response to opioids and associated with OUD.

Sexual dimorphism in ECM signaling pathways that regulate synaptic plasticity and neuroinflammation has been found in fish, birds, mice, and humans. In zebrafish, gene expression patterns for genes associated with the production of ECM signaling proteins are overrepresented in males (Wong et al., 2014), while sex differences in the number and formation of PNNs are observed in zebra finches (Cornez et al., 2015). Sex specific transcriptomic differences are found in mouse sensory neurons, specifically in genes related to neurotransmission, inflammation, and ECM reorganization, suggesting potential sex differences in susceptibility to neuroinflammation and ECM (Mecklenburg et al., 2020; Batzdorf et al., 2022) and OUD (Cahill and Taylor, 2017; Jang et al., 2020). In humans, differences in ECM signaling markers and remodeling are also found in blood serum depending on age and sex, irrespective of disease-related factors (Kehlet et al., 2018). While these few studies on sex-specific effects in ECM are sparse, they are particularly relevant considering the sexual heterogeneity in OUD.

## Conclusion

In this review, we highlight the current understanding of the interactions between ECM signaling, neuroinflammation, and synaptic plasticity, as it contributes to opioid seeking, craving, and relapse behaviors. Overall, there is a need for additional research investigating the potential role for biological sex at the intersection of ECM signaling and remodeling, synaptic plasticity, neuroinflammation, and opioids. Targeting specific ECM signaling proteins (e.g., MMPs and CAMs) during opioid administration and/or withdrawal could be a viable therapeutic approach. Preclinical models of opioid self-administration, opioid tolerance, and withdrawal, as well as pain and analgesia, provide tractable approaches that can provide depth into the potential roles of the ECM in opioid-related neurobiology and behavior. The inclusion of sex as a biological variable in these studies should aid in the discovery of novel therapeutic targets for the treatment of opioid dependence and OUD, while also supporting more inclusive options for interventions and therapeutics.

## Author Contributions

MHR, BRW, MKK, CDB, and RWL conducted a review of the literature and wrote the manuscript. All authors contributed to the article and approved the submitted version.

## Conflict of Interest

The authors declare that the research was conducted in the absence of any commercial or financial relationships that could be construed as a potential conflict of interest.

## Publisher’s Note

All claims expressed in this article are solely those of the authors and do not necessarily represent those of their affiliated organizations, or those of the publisher, the editors and the reviewers. Any product that may be evaluated in this article, or claim that may be made by its manufacturer, is not guaranteed or endorsed by the publisher.
